# Cost-effectiveness of interceptive orthodontics: a long-term evaluation of early treatment strategies

**DOI:** 10.1093/ejo/cjag022

**Published:** 2026-04-27

**Authors:** Sara Björns, Klara Leffler, Thomas Davidson, Anna Westerlund

**Affiliations:** Department of Cariology, Institute of Odontology, Sahlgrenska Academy, University of Gothenburg, Box 450, 405 30 Gothenburg, Sweden; Department of Preventive and Community Dentistry, Public Dental Service, Region Västra Götaland, Public Dental Service Västra Götaland, Box 7163, 402 33 Gothenburg, Sweden; Department of Mathematical Sciences, University of Copenhagen, Universitetsparken 5, 2100 Copenhagen, Denmark; Department of Health, Medicine and Caring Sciences, Linköping University, 581 83 Linköping, Sweden; Department of Orthodontics, Institute of Odontology, Sahlgrenska Academy, University of Gothenburg, Box 450, 405 30 Gothenburg, Sweden

**Keywords:** health economics modeling, interceptive care, orthodontics, dental appliance

## Abstract

**Objectives:**

Malocclusions occur at high frequencies in children and adolescents. While early orthodontic (interceptive) interventions may reduce the need for later comprehensive treatment, their cost-effectiveness in publicly funded health systems is unclear. This study compares the cost-effectiveness of interceptive orthodontic care and fixed-appliance therapy, focusing on total costs, treatment outcomes, and resource use.

**Methods:**

A decision tree model was developed using observational data on treatment success rates, duration, and appointments to estimate resource use and costs for interceptive orthodontic care; Fixed Appliance therapy was modeled as an optimal 2-year treatment pathway. A health-care payer perspective was applied. Incremental costs and effects were calculated for four interceptive modalities: Quad Helix, Extraoral Traction (EOT), Removable Plates, and Activator appliances, as compared to fixed appliance therapy. Minimum required success rates were estimated for achieving cost-neutrality. Probabilistic sensitivity analyses (10 000 Monte Carlo simulations) and scenario analyses assessed the robustness of the results.

**Results:**

All the interceptive treatments demonstrated lower expected costs than the fixed appliance therapy, albeit with lower clinical effectiveness. Quad Helix exceeded the minimum required success rate 20% points, representing a clear margin of cost-effectiveness. Removable Plates also exceeded the minimum required success rate, but with a smaller margin of 6% points. The Activator and EOT appliances were more dependent on patient compliance and failed to meet the minimum required success rate, requiring increases of 13% and 10% points, respectively, to achieve cost-neutrality. Sensitivity analyses confirmed these patterns and underscored the importance of long-term treatment stability.

**Limitations:**

The model assumed a 100% success rate for fixed appliances and relied on expert opinion for long-term stability parameters, given the limited availability of relapse data.

**Conclusions:**

In the publicly funded dental care context studied, the use of Quad Helix and removable plates in publicly funded health-care systems appears to be cost-effective. Activator and EOT appliances should be used selectively. The study also contributes a transparent, adaptable modeling framework that can incorporate locally relevant costs and future long-term outcome data, supporting use in other publicly funded settings.

## Introduction

Malocclusion occurs in ∼70% of children and adolescents during craniofacial development, with adverse effects on oral health-related quality of life, psycho-social well-being, and self-esteem [1, 2]. Children with significant malocclusions often present with severe social anxiety and diminished self-esteem [3–5]. In Sweden, dental care is publicly funded for all individuals under the age of 19 years, with the primary objective of preserving oral health through the prevention and timely management of disease. This includes monitoring tooth eruption and dental development over time, as well as intercepting and correcting malocclusions with orthodontics when they become clinically severe, so as to maintain function, support esthetics, and promote psycho-social well-being.

Orthodontic treatment follows two main pathways: early interceptive treatment during the mixed dentition phase; and comprehensive treatment of the permanent dentition. Interceptive strategies aim to guide dental development, employing simple appliances over shorter periods to achieve limited objectives [6–8]. In contrast, comprehensive treatment with fixed appliances seeks ideal occlusion but requires more time and technical precision and carries greater risks, such as appliance-related side-effects, and its long-term success depends on effective retention [6–8].

Earlier studies on effectiveness focused on either two-phase (early and late) or single-phase treatments initiated in the permanent dentition, and they often concluded that a single-phase approach was the most-cost-effective [9–12]. This is largely based on the assumption that the goal is to achieve a final, ideal occlusal result. In publicly funded health-care systems, however, where the objective is to deliver treatment to as many patients as possible with limited resources, the roles and value of interceptive treatments have not been thoroughly investigated. This is partly due to the conventional definition of treatment success, typically based on ideal outcomes, and partly due to the limited availability of long-term follow-up data for early interventions.

Other studies have investigated the clinical effectiveness and cost implications of early orthodontic interventions using various interceptive approaches, but not comprehensive fixed appliances. Kallunki *et al*. [13] conducted a randomized controlled trial designed to compare headgear Activator treatment initiated in the mixed versus late mixed dentition, reporting no significant differences in costs or treatment outcomes. Between the two approaches, though highlighting the high percentages of unsuccessful outcomes in both groups (27%–29%) and the need for subsequent fixed appliance therapy in several cases. Petrén *et al*. [14] demonstrated that Quad Helix treatment for unilateral posterior crossbite was associated with lower overall costs and fewer failures than expansion plates, further supporting its use in interceptive orthodontics. Sollenius *et al*. [15] compared Quad Helix and expansion plate treatments for posterior crossbite across specialist and general dental settings. Their findings revealed the superior clinical outcomes and lower societal costs attained when orthodontic specialists administered Quad Helix. Similarly, Wiedel *et al*. [16] reported that fixed appliances for anterior crossbite were more cost-efficient than removable plates due to lower material and indirect costs, despite comparable clinical outcomes. Taken together, these studies highlight the variability in cost and outcome profiles among interceptive appliances, which the variations depending on timing of the intervention, provider type, and appliance design. However, few analyses have integrated real-world regional data with long-term health economics modeling to estimate incremental cost-effectiveness across multiple interceptive modalities. This study addresses that gap.

In a recently large-scale cohort study of interceptive orthodontics in Region Västra Götaland of Sweden [17], treatment outcomes were analysed for more than 21 000 interceptive treatments performed between 2020 and 2024. The study included four commonly used appliances, Quad Helix, EOT, Removable Plate, and Activator, with success rates ranging from 56% to 82%. The dataset was derived from a publicly funded health-care system encompassing ∼400 000 children and adolescents aged 0–19 years. Each year, care was delivered through more than 100 public dental clinics by ∼600 general dentists, in close collaboration with 12 specialist orthodontic units and 40 orthodontic consultants. This unique regional infrastructure, with centralized specialist support and a structured referral system, allows for uniform access to interceptive care across a broad population base. The findings reflect real-world treatment conditions in general practice. Despite this breadth of clinical insight, the economic implications of these differences, both in terms of direct health-care costs and their potentials to reduce subsequent needs for comprehensive fixed appliance therapy, have not yet been evaluated.

Cost-effective allocation of interceptive orthodontic care could help to reduce the burden of more-complex treatments later in life. This study builds upon previous clinical findings by modeling the cost-effectiveness of commonly used interceptive appliances compared with fixed appliance therapy from the perspective of a publicly funded dental care system. The aim of this study is to evaluate the cost effectiveness of four interceptive modalities: Quad Helix, EOT, Removable Plates, and the Activator compared with fixed appliance therapy.

## Materials and methods

### Study design and perspective

A health economics modeling [18] approach was employed, and a decision tree model [19] was used to map out the potential outcomes and costs associated with each treatment option. The modeling approach adhered to the ISPOR and CHEERS 2022 guidelines [20]. The study adopted the perspective of the regional health-care payer, i.e. the Public Dental Service. This perspective aims to provide insights that can inform policy decisions related to resource allocation within the region’s publicly funded dental care system. The study was approved by the Swedish Ethical Review Authority (Dnr. 2024-01395-01).

### Population and data sources

The study cohort consisted of children in the age range of 6–14 years who initiated orthodontic treatment in Year 2020 (*N* = 8833). Of these, 4745 received interceptive therapies (Quad Helix, EOT, removable plate, or Activator), and 4088 received fixed appliance treatment. In Region Västra Götaland, interceptive orthodontic treatments are primarily delivered by general dentists within Public Dental Service clinics. Removable plates are typically used for single-tooth anterior crossbite, posterior crossbite, large overjet with proclined incisors aimed at achieving competent lip closure, and deep bite with gingival impingement, consistent with the indications reported in the previous large-scale cohort study [17].

For technique-sensitive appliances and more-complex cases, structured collaboration with regional orthodontic specialists forms part of routine care, including case discussions and treatment planning support. Referral to comprehensive fixed appliance therapy is determined after reassessment against established regional eligibility criteria. This organizational structure underpins the decision pathways modeled in the present analysis. A schematic overview of the care pathway is provided in Supplementary Figure S1. Data on clinical effectiveness (e.g. treatment success rates) were observational data obtained from regional clinical databases [17] (Table 1). Theoretical assumptions informed the probabilities of transitioning to subsequent treatment modalities. Cost data, encompassing materials, professional fees, treatment durations, treatment appointments and overhead expenses, were compiled using unit costs (materials, professional fees, overhead) extracted from the Public Dental Service’s administrative databases for year 2025. Treatment durations and appointments were obtained from the previously described large-scale regional cohort of interceptive orthodontic treatments. Costs were reported in Swedish Krona (SEK) and converted to Euro (€) using the average exchange rate for 2024 (1SEK = €0.0875) [21]. All costs are summarized in Supplementary Table S1.

**Table 1:** Observed treatment success rates for each intervention, based on patient data from registered treatments for children aged 6–18 years in Region Västra Götaland during 2020 [19].

Treatment	Initial cases (N)	Success cases	Uncertain (partial success) cases	Failure cases
Fixed appliances	4000	4000 (100%)	—	—
Removable plate	1764	1145 (64.9%)	484 (27.4%)	149 (7.7%)
Quad helix	655	538 (82.1%)	57 (8.7%)	60 (9.2%)
Extra-oral traction	101	58 (57.4%)	9 (8.9%)	34 (33.7%)
Activator	1327	746 (56.2%)	184 (13.9%)	397 (29.9%)

The table reports the number of patients and corresponding success, partial success, and failure rates used to inform the decision-analytic model. These values served as input parameters for both the base case and sensitivity analyses.

### Decision tree model

**Figure 1:**
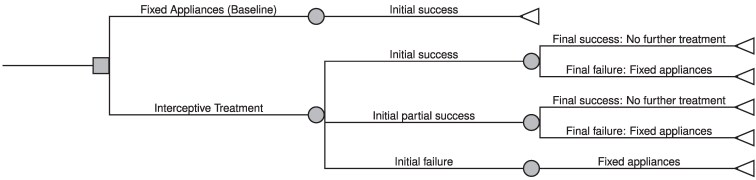
Decision tree showing treatment pathways and outcome, illustrating the possible sequences of initial success, partial success, or failure, and the resulting need for further treatment with fixed appliances. Final outcomes reflect either long-term success without further treatment or failure requiring continued intervention.

The decision tree structure was designed to reflect the logical clinical pathway for patients who were under consideration for interceptive treatment. The structure includes the pathways and possible outcomes associated with interceptive and fixed appliance treatments. Comparators were selected based on the most commonly used interceptive appliances in routine practice and the standard fixed appliance therapy for malocclusions that required correction. Each initial branch in the tree represents a possible decision point, leading to different outcomes in terms of effectiveness and potential follow-up treatment requirements. For patients receiving interceptive treatment, we considered three possible initial treatment outcomes: success, partial success, and failure. Partial success was defined as a clear but incomplete correction of the primary treatment indication for the respective appliance. For instance, following quad helix treatment, this corresponded to partial correction of the posterior crossbite, with residual transverse discrepancy remaining. Appliance-specific outcome definitions have been described previously [17]. Treatment success was defined based on whether the interceptive intervention reduced malocclusion severity below the regional eligibility threshold for publicly funded comprehensive orthodontic treatment. Standardized PAR score recordings were not consistently available in the registry dataset. Given that the economic model was structured around avoidance of subsequent fixed appliance therapy, this decision-based threshold was considered more directly relevant than continuous occlusal indices. Treatment failures were those cases in which patients were assessed as needing future follow-up treatment with fixed appliances. We also considered two long-term outcomes: success and failure. The initial treatment outcomes reflect the levels of success of the initial corrections, while the long-term outcomes reflect whether continued treatment with Fixed Appliances will be needed. Therefore, any initial successful treatment might still result in a relapse with a need for follow-up treatment. In this study, we only consider one type of follow-up treatment, a namely fixed appliance, which is assumed to have a 100% success rate. A graphical representation of the decision tree model is presented in Fig. 1.

### Costs and effects

The costs for each treatment modality were calculated by including direct medical costs, such as materials, dental professional fees, and overhead. Personnel and overhead costs were calculated based on treatment duration, number of visits, and chair-time per visit. The direct medical costs were derived from annual standardized cost schedules and unit prices. Data on treatment duration and number of visits were obtained from the previously described large-scale regional cohort of interceptive orthodontic treatments. Chair-time per visit was estimated based on established clinical routines. For Fixed Appliances, treatment duration and resource use were additionally based on an assumed ideal treatment length of 2 years, in accordance with standard clinical routines. Supplementary Table S1 shows a detailed summary of all the considered costs.

Treatment effectiveness was evaluated as the rate of success for an initial treatment outcome. Treatment outcomes were derived from the same regional cohort dataset of interceptive orthodontic care. The success rates of the initial treatments were estimated from the data in the regional clinical database, and they served as pathway probabilities in the decision tree model (Table 2). Since the long-term stability effects have yet to be assessed for the considered cohort, experts in the field set reasonable rates for the end-nodes.

**Table 2:** Input parameters for the decision-analytic model.

					Final outcomes			
		Initial treatment outcomes			After initial success		After initial partial success	
Treatment	Cost (€)	Success	Partial success	Failure	Success	Failure	Success	Failure
Fixed appliance	2501	1	0	0	1	0	0	0
Removable plate	1144	0.649	0.274	0.077	0.75	0.25	0.5	0.5
Quad helix	1254	0.821	0.087	0.092	0.75	0.25	0.5	0.5
Extra-oral traction	994	0.574	0.089	0.337	0.75	0.25	0.5	0.5
Activator	1152	0.562	0.139	0.299	0.75	0.25	0.5	0.5

Treatment costs and initial treatment outcomes (success, partial success, and failure rates) were based on data from registered treatments in Region Västra Götaland during 2022. Final treatment outcome probabilities following initial success or initial partial success were informed by clinical assumptions and expert opinion. The Fixed Appliance group is assumed to achieve full success with no subsequent failure, serving as the baseline comparator.

### Cost-effectiveness analysis

The Cost-effectiveness analysis (CEA) was based on the expected cost for each treatment and the cost per unit of treatment success for each interceptive treatment modality, as compared to the corresponding data for the fixed appliance therapy. The expected cost was defined as the total cost per patient for a fully completed pathway through the model, i.e. for either a fixed appliance therapy or any interceptive treatment followed by a subsequent fixed appliance therapy, if needed. We computed the expected cost as the weighted sum across all pathways of each pathway cost multiplied by its respective pathway probability, as follows:


Expectedcost=∑(pathway×pathwaycost).

**Figure 2:**
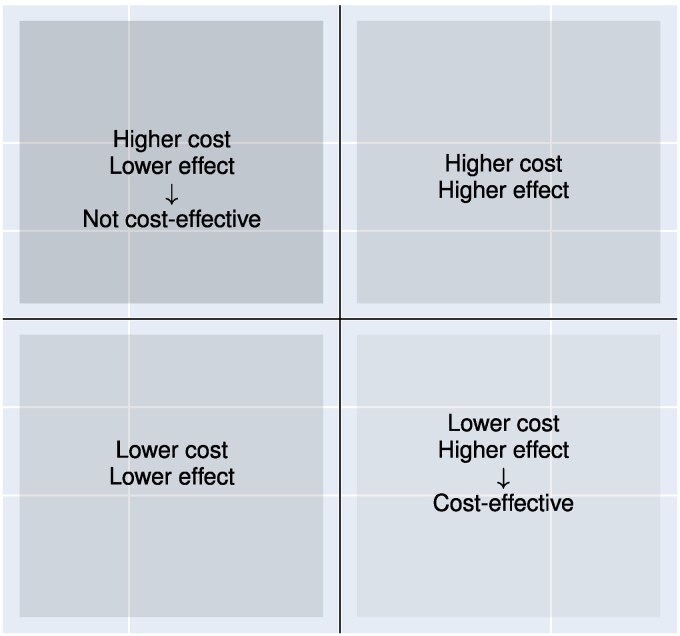
Visual representation of the cost-effectiveness plane and the interpretation of its quadrants: (1) northeast—higher cost and higher effect than the baseline; (2) northwest—higher cost and lower effect; (3) southeast—lower cost and higher effect; and (4) southwest—lower cost and lower effect. Alternatives are considered relative to a baseline in terms of their cost and effect. The Southeast quadrant represents the preferred option, while the Northwest quadrant is undesirable. Interventions in the Northeast and Southwest quadrants require tradeoff assessment, typically based on a WTP threshold, to determine whether they are preferred to the baseline.

From the expected costs, we calculated the incremental expected cost between the interceptive treatments and the fixed appliance treatments. The cost-effectiveness value can then be derived by dividing the incremental cost by the corresponding incremental effect, representing the additional cost per unit of treatment success when comparing the interceptive treatment to the baseline. We did not explicitly derive the ICER values but instead visualized the ICERs in a cost-effectiveness plane. In this study, we considered interceptive treatments that are less costly, but also less effective than the baseline of fixed appliances. Therefore, all the interceptive treatments are expected to appear in the Southwest quadrant of the cost-effectiveness plane (Fig. 2).

The decision tree model aims to bridge the gap between what has been observed and what would be expected to happen over a long-term time horizon. In this case, a duration of 8 years was chosen to reflect the full course of the interceptive treatment and subsequent orthodontic treatment. Interceptive treatment typically begins at around 8–9 years of age and lasts ∼2 years. Fixed appliance treatment often starts at around 14 years of age and lasts for about 2 years, resulting in a total treatment period that covers the childhood age span of ∼8–16 years. Costs were discounted by 3% annually.

### Uncertainty analysis

Cohort models rely on mean input values that are derived from observational data. A key strength of the present study is the availability of large-scale observational data capturing short-term treatment outcomes in real-world clinical settings. However, as with all observational data, some uncertainty remains, particularly when estimates are used to inform model-based extrapolations beyond the observed follow-up period. Therefore, we conducted a two-step sensitivity analysis of the costs and effects.

First, we used a probabilistic sensitivity analysis (PSA) to account for the within-population variation. The PSA accounts for the uncertainty related to treatment success rates derived from observational data. In the base case analysis, these rates were treated as fixed point estimates. Here, we explored the impact of uncertainty by sampling each treatment’s outcome probabilities (success, partial success, failure) from a Dirichlet distribution centered on the observed outcome proportions. We set the dispersion so that the marginal standard deviation of the success probability was ∼0.05, corresponding to ∼95% of draws lying within ±0.10 of the observed success rate. The fixed-appliance treatment, assumed to have perfect effect, was not varied.

Second, deterministic sensitivity analysis (DSA), or scenario analysis, was used to explore the impacts of systematic changes. This analysis included potential additional costs arising from surplus visits and variations in the expert-defined, long-term success rates.

## Results

### Base case analysis

The model indicates that all interceptive treatments are associated with lower expected costs compared with fixed appliances, as shown in Table 3. The expected costs (in ascending order) were: €1497 for Removable Plate; €1545 for Quad Helix; €1686 for EOT; €1728 for Activator; and €2501 for Fixed Appliances. Each intervention resulted in negative incremental costs and adverse incremental effects. Consequently, all the interceptive treatments fell within the Southwest quadrant of the cost-effectiveness plane, reflecting tradeoffs between cost savings and lower treatment success rate.

**Table 3:** Base case cost-effectiveness results for all treatment strategies.

Treatment	Expected cost (€)	Incremental cost	Expected effects	Incremental effects	Required effectiveness
Fixed appliance	2501	—	1	—	1
Removable plate	1497	−1004	0.66	−0.35	0.60
Quad helix	1545	−955	0.82	−0.18	0.62
Extra-oral traction	1686	−815	0.57	−0.43	0.67
Activator	1728	−773	0.56	−0.44	0.69

Expected costs are presented in Euro (€) alongside incremental costs relative to the baseline (Fixed Appliance), expected effects, incremental effects relative to the baseline (Fixed Appliance). The required effectiveness corresponds to the minimum proportion of treatment success needed for an interceptive strategy to achieve cost-effectiveness parity with the baseline of Fixed Appliances.

We estimated the minimum required effectiveness for each interceptive treatment to provide equivalent value for money by calculating the ratio of its expected cost to that of the baseline. This reflects the principle that clinical benefit must be proportionate to relative expenditure, so as to maintain comparable cost-effectiveness. The calculated minimum required effectiveness values for the used appliances were ∼0.60 for Removable Plate; 0.62 for Quad Helix; 0.67 for EOT; and 0.69 for Activator. This highlights the level of success that each treatment would need to achieve in order to match the cost-effectiveness of the fixed appliances (Table 3).

### Probabilistic sensitivity analyses

**Figure 3:**
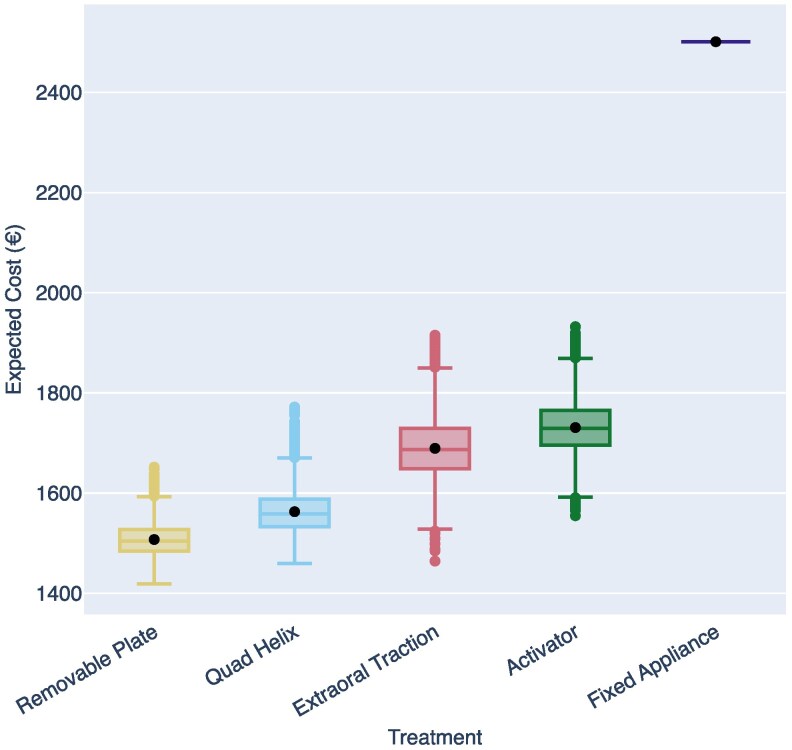
Distribution of expected costs for each treatment option across 10 000 Monte Carlo simulations in the PSA, illustrating how uncertainty affects expected treatment costs.

Model outputs were estimated over an 8-year time horizon using 10 000 Monte Carlo simulation iterations. The resulting mean expected costs (in ascending order) were: €1507 for Removable Plate, €1563 for Quad Helix, €1689 for EOT, €1731 for Activator, and €2501 for Fixed Appliance. The results are illustrated in Fig. 3, showing the distribution of the expected costs across simulations. Supplementary Table S2 summarizes the corresponding mean expected costs and effects, along with the standard errors.

**Figure 4:**
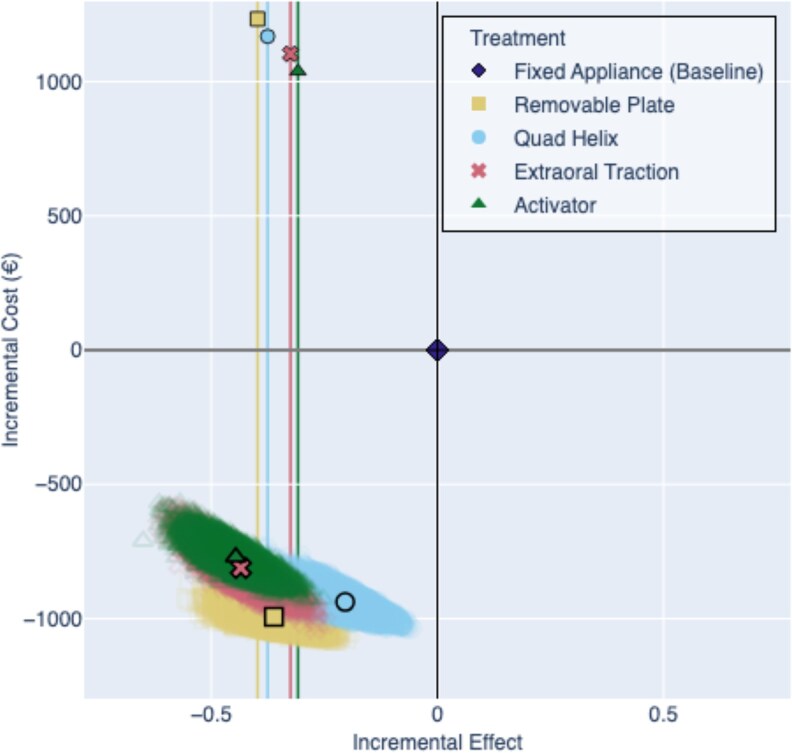
Cost-effectiveness plane from the PSA, showing the distribution of incremental cost and effect pairs for each interceptive treatment compared with the baseline treatment with fixed appliance. Each point represents one of 10 000 Monte Carlo simulations, and mean values are indicated by black outlines. The vertical lines represent the mean minimum required effectiveness for each interceptive treatment, i.e. the level at which the treatment would offer equivalent value for money compared with the baseline.

In terms of cost-effectiveness, the PSA showed that all interceptive treatments fell within the Southwest quadrant of the cost-effectiveness plane, indicating they are both less costly and less effective than the baseline. Among these, Quad Helix and EOT clustered closest to the bottom-right corner of this Southwest quadrant, suggesting a more-favorable tradeoff between cost and effectiveness (Fig. 4 and Supplementary Figure S2).

### Deterministic sensitivity analyses

**Figure 5:**
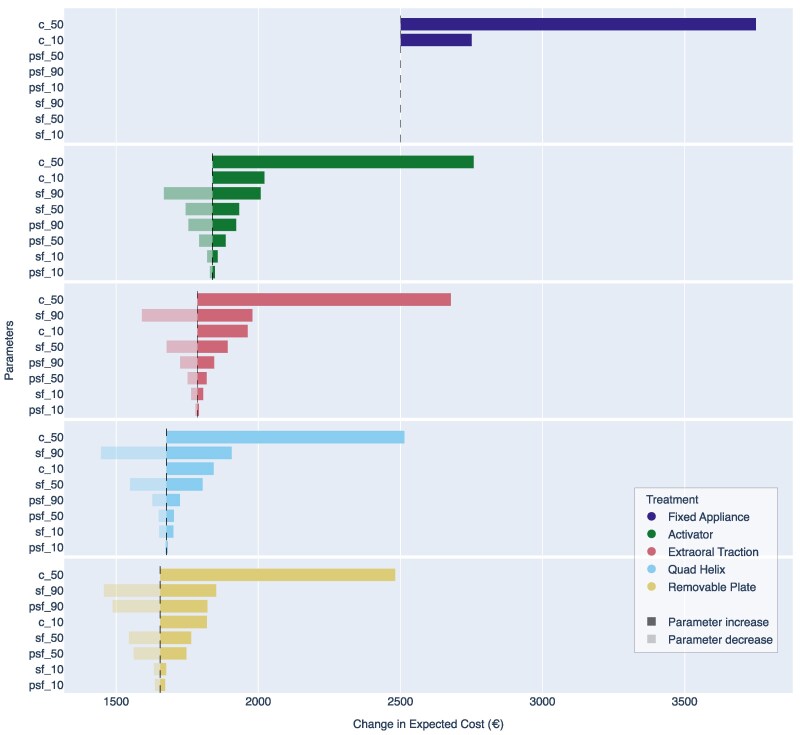
The effect of deterministic parameter variations on expected total costs. Dashed lines represent base case values, while parameter variations are shown as colored bars. Each bar represents the change in expected cost resulting from varying a single parameter while holding others constant. Parameters include treatment cost increases (10% and 50%) and variation of long-term failure rates (10%, 50%, and 90%) in both favorable and unfavorable directions. Parameter abbreviations should be interpreted as the given number in percentage change and: c = cost, sf = failure rate after initial success, and psf = failure rate after initial partial success.

To evaluate the impacts of systematic changes on treatment costs and long-term outcomes, we conducted a DSA of the individual scenarios. The set-up reflects the short- to medium-term economic variations. Cost parameters were varied at two levels: a 10% and a 50% increase, simulating increasing resource use or overheads. The long-term failure rate, defined as the proportion of patients requiring fixed appliances after interceptive treatment, was varied at 10%, 50%, and 90% in both directions to reflect favorable and unfavorable outcomes. The most-influential factor was a substantial cost increase, which strongly affected the total expected costs. For the most-cost-effective interceptive option (Quad Helix), variation of the long-term failure rates also significantly affected the model outcomes. Figure 5 summarizes the effect of each parameter variation on the expected total costs. Details of the DSA inputs are summarized in Supplementary Table S3. The primary outcome was treatment success, defined as clinically acceptable occlusion without the need for follow-up fixed appliance therapy.

## Discussion

Even though studies have shown that single-phase treatment with fixed appliances is the most efficient way to achieve an ideal treatment outcome, publicly funded systems with finite budgets face an inherent tradeoff: whether to allocate resources so that a relatively small number of patients can achieve an ideal result, or to treat a larger number of children to reduce their malocclusion below the threshold at which they qualify for publicly funded care (in our context, roughly IOTN grades 4–5), with any further refinement being self-funded. In such settings, early interceptive treatment has often been assumed to be more cost-effective, and similar reasoning may apply to patients with limited financial means in other health-care systems. This large-scale, real-world economic evaluation of interceptive orthodontic strategies in Sweden’s publicly funded dental system demonstrates that while all interceptive appliances incur lower direct costs than fixed appliance therapy, they also exhibit reduced clinical effectiveness. Among the options, the Quad Helix appliance is the most favorable, having the highest success rate (82%) and the second lowest mean cost. In contrast, the Activator and EOT exhibit lower levels of effectiveness and poorer economic performance, likely influenced by their heavy reliance on patient compliance.

The Region Västra Götaland delivers publicly funded dental care to ∼400 000 individuals in the age range of 0–19 years. Over the 5-year period studied, more than 21 000 interceptive orthodontic treatments were registered across 600 general dentists (working at 103 clinics), supported by 40 orthodontic consultants (across 12 specialist clinics). This comprehensive treatment infrastructure enabled large-scale delivery of the Quad Helix, EOT, Removable Plate, and Activator appliances. While the economic evaluation was based on the full treatment dataset, treatment success was assessed through an in-depth manual review of about 4000 patient records. This dual-level approach offers both breadth and analytical depth, making this study the largest real-world assessment of interceptive orthodontics in a publicly funded health-care setting conducted to date [17].

The base-case and probabilistic sensitivity analyses both demonstrate that all the interceptive treatments are associated with lower direct costs compared with Fixed Appliance therapy. However, this economic advantage is diminished by lower clinical effectiveness, placing all interceptive options within the Southwest quadrant of the cost-effectiveness plane, being less costly but also less effective than the fixed appliance (Fig. 4). Among these, Quad Helix and Removable Plate offer the most-favorable tradeoffs, with Quad Helix emerging as the most-cost-effective option, driven by its high success rate. In contrast, the Activator and EOT treatments show lower levels of cost-effectiveness. The Activator appliance has the highest expected mean cost and the lowest success rate. These findings align with those of previous studies [14, 15], which reported superior clinical outcomes and reduced societal costs when Quad Helix was used to treat posterior crossbite, particularly in specialist care settings. Although Petrén and coworkers did not include a direct comparison with Fixed Appliance therapy, their analysis showed that Quad Helix was more cost-effective than Removable Expansion Plate in managing unilateral posterior crossbite. It is important to note, however, that their study focused exclusively on expansion as the indication for treatment. The present study encompasses a broader range of indications for Removable Plate, extending beyond transverse discrepancies. Removable Plate also demonstrates cost-effectiveness, with moderate costs and success rates (65%) [17]. However, their effectiveness is highly dependent upon full-time wear and a high level of patient motivation. Previous findings [16] have suggested that although plates and Fixed Appliances yield similar clinical outcomes, the former are more sensitive to compliance and may incur higher indirect costs in general practice, due to treatment failures and follow-up demands.

The Activator and EOT, both of which are part-time appliances, are less-cost-effective under the current parameters. The Activator shows the highest expected cost and the lowest success rate (56%) of all the interceptive appliances studied. The finding that the Activator is not cost-effective, despite its widespread use, highlights an important drawback of this treatment approach. Given the high prevalence of pronounced overjet among children and its documented association with increased risk of bullying, psycho-social distress, and reduced self-esteem, early treatment remains a clinical priority [22, 23]. Functional appliances, such as the Activator, are intended to address these concerns within a window of dentoalveolar adaptability. However, their success is critically dependent upon patient compliance. The low compliance observed in this cohort highlights a key barrier that must be addressed. These appliances are inherently technique-sensitive, and the Activator, extraoral traction, and removable plates are likewise strongly dependent on patient co-operation, which contributes to substantial variation in outcomes. In our material, just over half of the treatments were successful, and treatment effectiveness was entirely driven by the level of patient compliance. For this reason, their use should be limited to cases where good co-operation can reasonably be expected. Successful outcomes also presuppose that the appliance is used in patients who have reached an appropriate stage of treatment maturity, both in terms of growth and dental development, and who are psychologically mature enough to manage the demands of treatment. Based on current treatment costs, Activator therapy must achieve a success rate of at least 69% in order to be as cost-effective as the fixed appliances; this success rate has been demonstrated by Kallunki *et al*. [13] but has rarely been reported elsewhere. These findings reinforce the importance of strategies to enhance compliance. Future strategies may include even-more-selective treatment approaches that target only highly motivated patients, as well as the implementation of digital monitoring tools to support adherence. These could include App-based wear-time tracking, remote follow-up, and gamified feedback systems that engage both patients and parents. Moreover, alternative Class II appliances with higher efficacies should be considered. The performance of Removable Plate (success rate of 65%) suggests that full-time wear plays a crucial role in achieving stable outcomes. Appliances such as the Twin Block, which are designed for near-continuous wear and have shown favorable outcomes in clinical trials, may offer higher cost-effectiveness compared with the Activator device [11]. Regarding EOT, current clinical trends within the region already reflect a move away from its use, particularly for space-gaining purposes prior to canine eruption. Instead, premolar extractions have become a more-common strategy. This shift appears justified given the relatively low cost-effectiveness of EOT, combined with its high failure rate and reliance on part-time wear.

As with all cost-effectiveness analyses, the interpretation of outcomes is influenced by the underlying assumptions and parameter settings of the model. In the present study, success was determined based on appliance-specific clinical outcomes aligned with the threshold for publicly funded care (IOTN > 3), which represents a moderate-to-severe malocclusion.

Treatment outcomes are highly dependent upon patient co-operation, particularly for removable and part-time appliances, as well as on the complexity of the malocclusion and the patient’s growth trajectory. In our cost model, both interceptive treatments were costed solely on the outcome from the large-scale cohort study [17] on treatment duration and number of visits; emergency appointments were not included because the data were less robust. Fixed appliances were assigned a 100% success rate as an idealized reference, reflecting their technical capacity to fully correct the malocclusion, in contrast to interceptive approaches, which generally have more limited treatment goals. Variables such as visit intervals, chair-time allocation, and definitions of treatment success can be adjusted within the model to reflect different clinical protocols and health-care priorities. In addition, Fixed Appliance therapy was assumed to yield a 100% success rate, which may overstate its real-world effectiveness. Unfortunately, long-term follow-up data on relapse and re-treatment were not yet available for the interceptive cohort. While there is a temporal gap between the sources of the pathway probabilities (Year 2020) and the cost data (Year 2025), expert opinions received from orthodontists suggest that clinical practice and patient flow patterns have remained essentially unchanged since 2020. This supports the continued relevance of the Year 2020 data. Nevertheless, future models should incorporate long-term outcome data and sensitivity analyses of relapse probabilities to estimate with greater accuracy real-world effectiveness. A key limitation of the present study, as well as of most other cost estimations of care, is the extent to which its findings can be generalized beyond the setting from which the data were derived. Clinical outcomes, patterns of care, and cost structures in interceptive orthodontics are inherently context-dependent, varying across health-care systems, reimbursement models, and organizational frameworks. Our cost analysis is based on a large, real-world epidemiological dataset comprising ∼30 000 interceptive orthodontic treatments over a 5-year period, of which nearly 5000 cases were examined in detail. These data reflect routine care delivered within a publicly funded dental service, and the estimates presented here should therefore primarily be interpreted within that context. We expect the impact of the care model to be most pronounced for operator-sensitive appliances. In this regard, the Quad Helix is likely to be the most technique-dependent modality, requiring skill in band selection, cementation, and activation [15]. For the other three appliances in the present study, lack of success was predominantly due to nonuse or insufficient use of the appliance rather than technical failures. While it is possible that a specialist-led service with stronger behavioral support (?) might influence adherence and thereby effectiveness, robust comparative data are currently lacking.

The organizational features of this system, including comprehensive population coverage and standardized treatment pathways, are shared with other publicly financed dental services in Europe. For clinicians and policy makers working in similar environments, the present results may therefore provide useful benchmarks for the relative resource implications of different interceptive strategies. In settings with substantially different fee structures, case mixes, or models of care delivery, however, direct extrapolation of our cost estimates requires caution. In such contexts, our findings are best regarded as a structured reference point that can be adapted and recalibrated to local conditions rather than as directly transferable absolute figures. The ability to adapt the analysis to local conditions follows from the structure of the decision model itself, which is built around three modular input categories: empirically observed total costs, short-term success rates derived from a large, real-world epidemiological dataset, and provisional expert-derived long-term stability estimates. Each of these components addresses a distinct aspect of the decision problem and can be considered separately. Empirically observed total costs can be recalibrated to reflect local cost structures, staffing arrangements, and clinical protocols. The short-term success rates, derived from a large population-based cohort under routine clinical conditions, represent robust real-world effectiveness measures that should be relevant and applicable across other publicly funded or similarly organized health-care systems. The provisional expert-derived long-term stability estimates reflect clinically informed assumptions in the absence of long-term follow-up data and can be replaced by empirically observed outcomes as such data become available. Because these components can be modified independently, the model retains the same analytical logic while allowing adaptation to local conditions. In this way, the contribution of the present study is twofold: first, it provides a transparent and adaptable modeling framework that allows other health-care systems to estimate their own cost-effectiveness outcomes using locally relevant inputs; and second, it offers real-world empirical benchmarks from one of the largest population-based interceptive orthodontic datasets available, enabling meaningful comparison and scenario testing across different organizational contexts.

The scenario analyses in this study reveal that long-term treatment success rates are among the most-influential parameters, underscoring the importance of collecting long-term outcome data. The present analysis adopted a restricted provider perspective focusing on direct treatment costs and clinically documented resource use, because robust, generalizable data on patient- and family-related indirect costs are lacking. Consequently, our estimates do not represent full societal costs and should be interpreted accordingly. We restricted the analysis to direct treatment outcomes and costs and did not model potential long-term economic benefits of trauma prevention or other health gains from correcting malocclusions (e.g. crossbites, deep bites, crowding etc), as the current evidence base is insufficient for robust monetary valuation. Moreover, the cost-effectiveness results place all interceptive options in the Southwest quadrant, indicating reduced effectiveness, but also reduced cost. This contrasts with the more-common northeast positioning seen in most economic evaluations, whereby alternatives offer greater benefits at higher costs. The cost-effectiveness of the interceptive approaches relative to the fixed appliances can be established if a treatment demonstrates a cost-effectiveness value that is below an accepted willingness-to-pay (WTP) threshold, achieves equivalent effectiveness at a lower cost or balances reduced effectiveness with sufficiently lower costs. In this analysis, the primary justification for adopting interceptive treatment is economic benefit rather than clinical superiority. The variation in the appliance positioning on the cost-effectiveness plane also warrants attention. Quad Helix was most favorably positioned, whereas the Activator ranked lowest. These results may challenge conventional interpretations of WTP thresholds, which are often represented as linear. In practice, societal WTP for orthodontic interventions may be nonlinear, favoring cost minimization when the clinical gains are marginal. Further investigation is needed to determine the appropriate WTP thresholds for orthodontic outcomes. Finally, our approach addresses the gaps highlighted in recent reviews, such as that of Jermyn *et al*. [24], which underscores the scarcity of model-based economic evaluations in orthodontics and the methodological limitations of existing studies. The findings also have ethical and system level implications. Some interceptive options may fall below cost effectiveness thresholds yet deliver child centered benefits not captured by payer models. Selective use may relieve specialist capacity and shorten waiting times.

In summary, early treatment has often been assessed against ideal clinical outcomes [9-12]. These benchmarks may not reflect the priorities of publicly funded health-care systems. In such systems, where treatment is guided by severity and resource constraints, the value of interceptive orthodontics lies in achieving sufficient improvement to reduce future treatment needs. However, interceptive care still consumes health-care resources and must, therefore, be effective and achieve both short- and long-term goals to be justifiable. The results of this study support the use of the Quad Helix appliance and Removable Plates for crossbite corrections, including minor adjustments such as single-tooth anterior crossbites. Given current costs and outcomes, treatments involving the Activator and EOT appliances cannot be justified.

## Conclusion

In the publicly funded dental care context studied, the Quad Helix and Removable Plates appear to be cost-effective options for interceptive orthodontics in routine care. The value of using the Activator and EOT devices is questionable, as their success rates would need to increase by 13 and 10% points, respectively, to achieve cost-neutrality, and their use should be limited to carefully selected cases. Future studies should utilize this large-scale dataset to quantify the long-term effectiveness of orthodontic appliances by linking early outcomes to the subsequent need for fixed appliance therapy, regardless of whether this is due to failure, partial success or relapse.

Beyond these appliance-specific findings, the present study is based on a large, real-world dataset on interceptive treatment outcomes and provides a transparent decision-analytic model. This framework allows other health-care systems to generate their own cost-effectiveness estimates by inserting local cost data and updated long-term outcomes, supporting informed decision-making across diverse organizational settings.

## Supplementary Material

cjag022_Supplementary_Data

## Data Availability

The authors have read the journal’s requirements for reporting the data underlying this submission. The data used in this study consist of de-identified, routinely collected administrative and clinical data from the Public Dental Service in Region Västra Götaland and are subject to legal and ethical restrictions. Individual-level data cannot be made publicly available. Aggregated data supporting the findings, model structure, parameter assumptions, and analytic code are available from the corresponding author upon request.
