# Supplementary material for: Cost-effectiveness of interceptive orthodontics: a long-term evaluation of early treatment strategies
Source: Eur J Orthod. 2026 Apr 27;48(3):cjag022. doi: 10.1093/ejo/cjag022 (PMC13111488; doi:10.1093/ejo/cjag022)
Supplement: cjag022_Supplementary_Data [file cjag022_supplementary_data.pdf]

Supplementary material for

Cost-effectiveness of interceptive orthodontics: A long-term evaluation of early treatment strategies

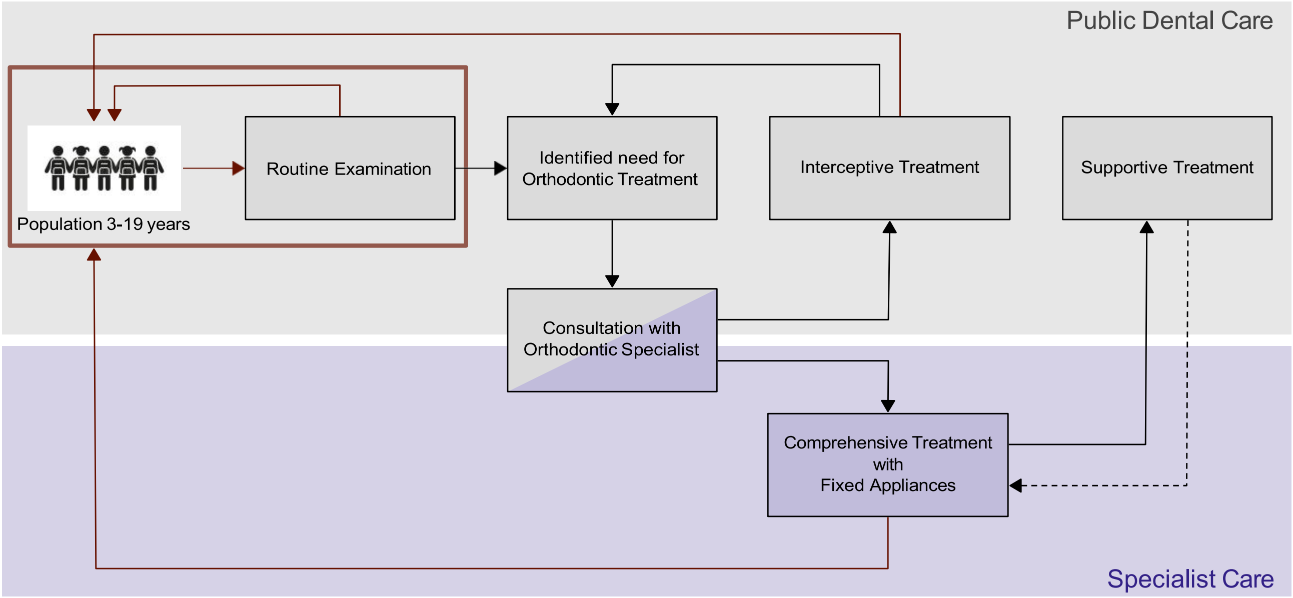

Figure S.1. Schematic illustration of the routine examination and recall pathway for individuals aged 3-19 years within general dental practice in Sweden (red box). Patients remain within this continuous loop with examinations and preventive care. If orthodontic treatment need is identified, they temporarily exit the loop to receive consultation with orthodontic specialist, followed by either interceptive care within general practice or Fixed Appliances at a specialist clinic. Following completion of orthodontic treatment, patients re-enter the general dental care recall pathway for ongoing monitoring and supportive care.

Table S.1. Breakdown of unit costs used as input to the decision model for each treatment strategy. The table includes all components contributing to the total cost per patient (e.g., appliance cost, clinical time, follow-up visits). The total costs shown in the rightmost column represent the values used as input to the decision tree model. Costs were reported in Swedish Krona (SEK) and converted to Euros (€) using the yearly average 2024 exchange rate (1 SEK = €0.0875).

Treatment	Treatment start		Treatment duration			Treatment end		Total time per occupation					Cost				
	Visits	Time *(min)*	Total time *(months)*	Visits	Time *(min)*	Visits	Time *(min)*	Dentist *(min)*	Nurse *(min)*	Orthodontic assistant *(min)*	Orthodontist *(min)*	Total *(min)*	Work hours *(SEK)*	Consult *(SEK)*	Lab *(SEK)*	Total *(SEK)*	Total *(€)*
Field scenario, based on Master Thesis data																	
Quad Helix	3	60	-	6.34	190	1	30	290	20	0	0	310	12081	952	1300	14333	1254
Extraoral Traction	2	60	-	8.21	164	1	30	234	20	0	0	254	9808	952	600	11360	994
Removable Plates	2	60	-	9.06	181	1	30	241	30	0	0	271	10226	952	1900	13078	1144
Activator	2	60	-	8.05	161	1	30	236	15	0	0	251	9937	952	2400	13167	1152
Ideal scenario, based on budget for each treatment																	
Fixed Appliances*	5	270	24	13[b]	390[c]	2	120	0	0	560	220	780	25643	952	2000	28595	2501
Quad Helix	3	90	12	6.6[a]	198[c]	1	30	298	20	0	0	318	12407	952	1300	14659	1282
Extraoral Traction	2	60	12	5.5[b]	110[d]	1	30	180	20	0	0	200	7599	952	600	9151	800
Removable Plates	2	60	15	10.8[a]	217[d]	1	30	277	30	0	0	307	11685	952	1900	14537	1272
Activator	2	60	24	9.5[b]	190[d]	1	30	265	15	0	0	280	10996	952	2400	14348	1255

*Note that there is no collected data for data for Fixed Appliance treatment in the field scenario, and that this study assumes an ideal scenario for Fixed Appliances.

[a]Check-up every 6 weeks.

[b]Check-up every 8 weeks.

[c]30 minutes per visit.

[d]20 minutes per visit.

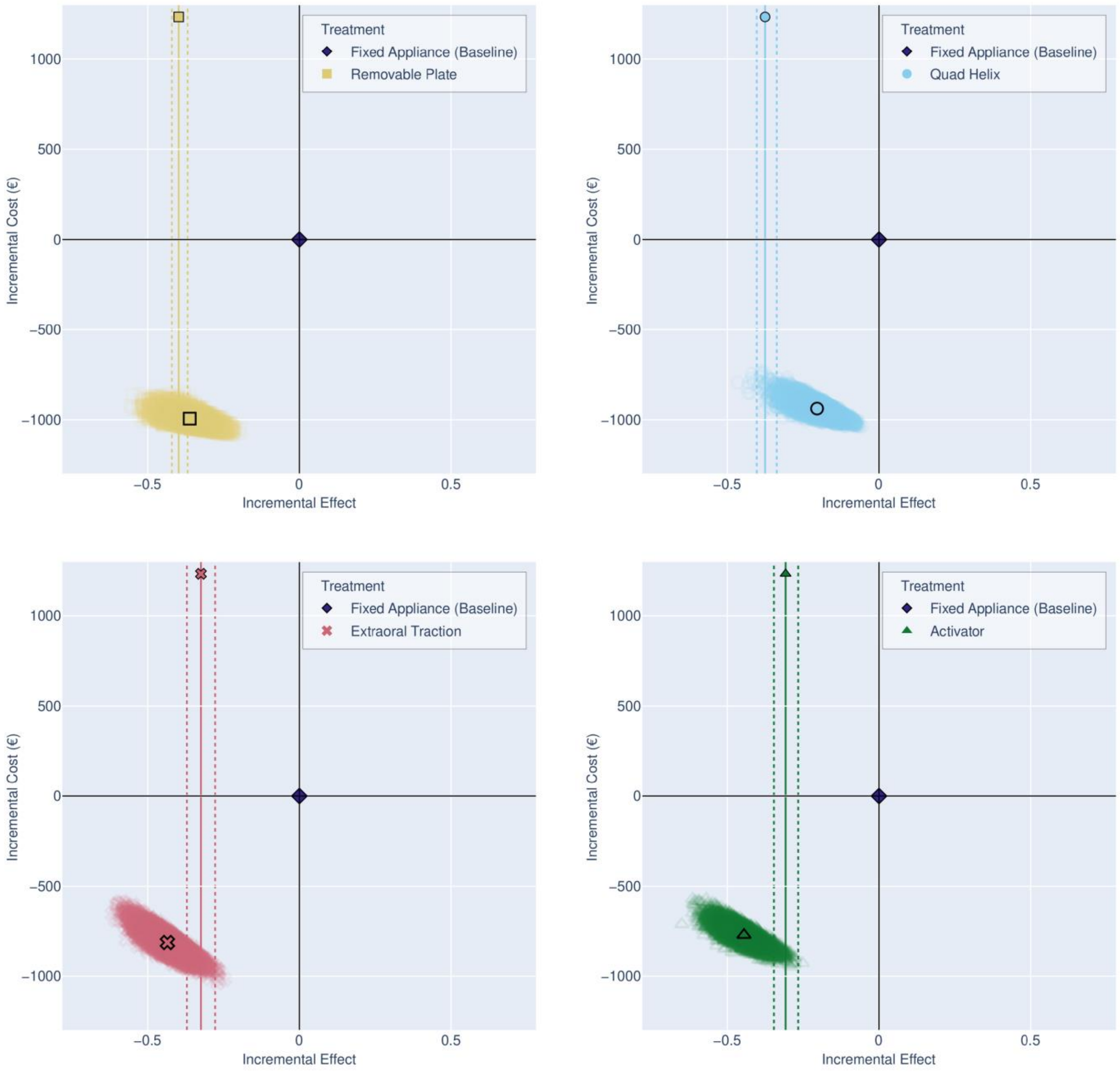

Figure S.2. Cost-effectiveness plane from the probabilistic sensitivity analysis, showing the distribution of incremental cost and effect pairs for each interceptive treatment compared to the baseline treatment with fixed appliance. Each point represents one of 10000 Monte Carlo simulations, and mean values are indicated by black outlines. The vertical lines represent the mean minimum required effectiveness for each interceptive treatment, i.e., the level at which the treatment offers equivalent value for money compared to the baseline. The dashed lines indicate the 95% empirical confidence interval for the minimum required effectiveness, based on the distribution of simulated values.

Table S.2. Probabilistic sensitivity analysis cost-effectiveness results for all treatment strategies. Mean values (and standard errors) are presented for expected costs, incremental costs relative to the baseline (Fixed Appliance), expected effects, incremental effects relative to the baseline (Fixed Appliance). The required effectiveness corresponds to the minimum proportion of treatment success needed for an interceptive strategy to achieve cost-effectiveness parity with the baseline of Fixed Appliance treatment.

Treatment	Expected cost (€)	Incremental cost	Expected effects	Incremental effects	Required effectiveness
Fixed Appliances	2501 (0.00)	-	1.00 (0.0000)	-	-
Removable Plates	1507 (0.34)	-994 (0.34)	0.64 (0.0005)	-0.36 (0.0005)	0.60 (0.0001)
Quad Helix	1563 (0.43)	-938 (0.43)	0.80 (0.0005)	-0.20 (0.0005)	0.62 (0.0002)
Extra-oral Traction	1687 (0.58)	-814 (0.58)	0.57 (0.0005)	-0.43 (0.0005)	0.67 (0.0002)
Activator	1730 (0.51)	-771 (0.51)	0.56 (0.0005)	-0.44 (0.0005)	0.69 (0.0002)

Table S.3. Parameter variations applied in the deterministic sensitivity analysis (DSA). This table presents the specific input values applied in each DSA scenario, including variations in treatment costs and long-term failure rates. Each parameter was varied individually while holding others constant. Treatment costs were specific to each intervention, resulting in different absolute changes across treatments. In contrast, failure rate scenarios were applied uniformly across all interceptive treatments and thus produced equal relative changes. As outcome probabilities must sum to 1, changes in failure rate also implied corresponding adjustments in final success rates. The failure rate for fixed appliances remained fixed at zero (i.e. 100% success). Initial success rates were not varied and are therefore omitted from this table.

Parameter	Treatment	Original parameter value	Parameter changes					
			–90%	–50%	–10%	+10%	+50%	+90%
Costs (€)	Fixed Appliances	2501				2751	3752	
	Removable Plates	1144				1258	1716	
	Quad Helix	1254				1379	1881	
	Extra-oral Traction	994				1093	1491	
	Activator	1162				1267	1728	
Final failure after initial success	Fixed Appliances	0.00	0.00	0.00	0.00	0.00	0.00	0.00
	Interceptive treatments	0.25	0.025	0.125	0.225	0.275	0.375	0.475
Final failure after initial partial success	Fixed Appliances	0.00	0.00	0.00	0.00	0.00	0.00	0.00
	Interceptive treatments	0.50	0.05	0.25	0.45	0.55	0.75	0.95
